# Supplementary material for: Asymmetric Synthesis of Quaternary Hydantoins via a Palladium-Catalyzed Aza-Heck Cyclization
Source: J Am Chem Soc. 2025 Nov 14;147(49):44692–8. doi: 10.1021/jacs.5c16022 (PMC12703750; doi:10.1021/jacs.5c16022)
Supplement: Supplementary file 2 [file ja5c16022_si_002.zip › All NMR FID Files/S8 2D Spectra/S8 Conformer 2 HMBC/8/pdata/1/email_EM06-035-isomer#2-1HMBC_8_1.pdf]

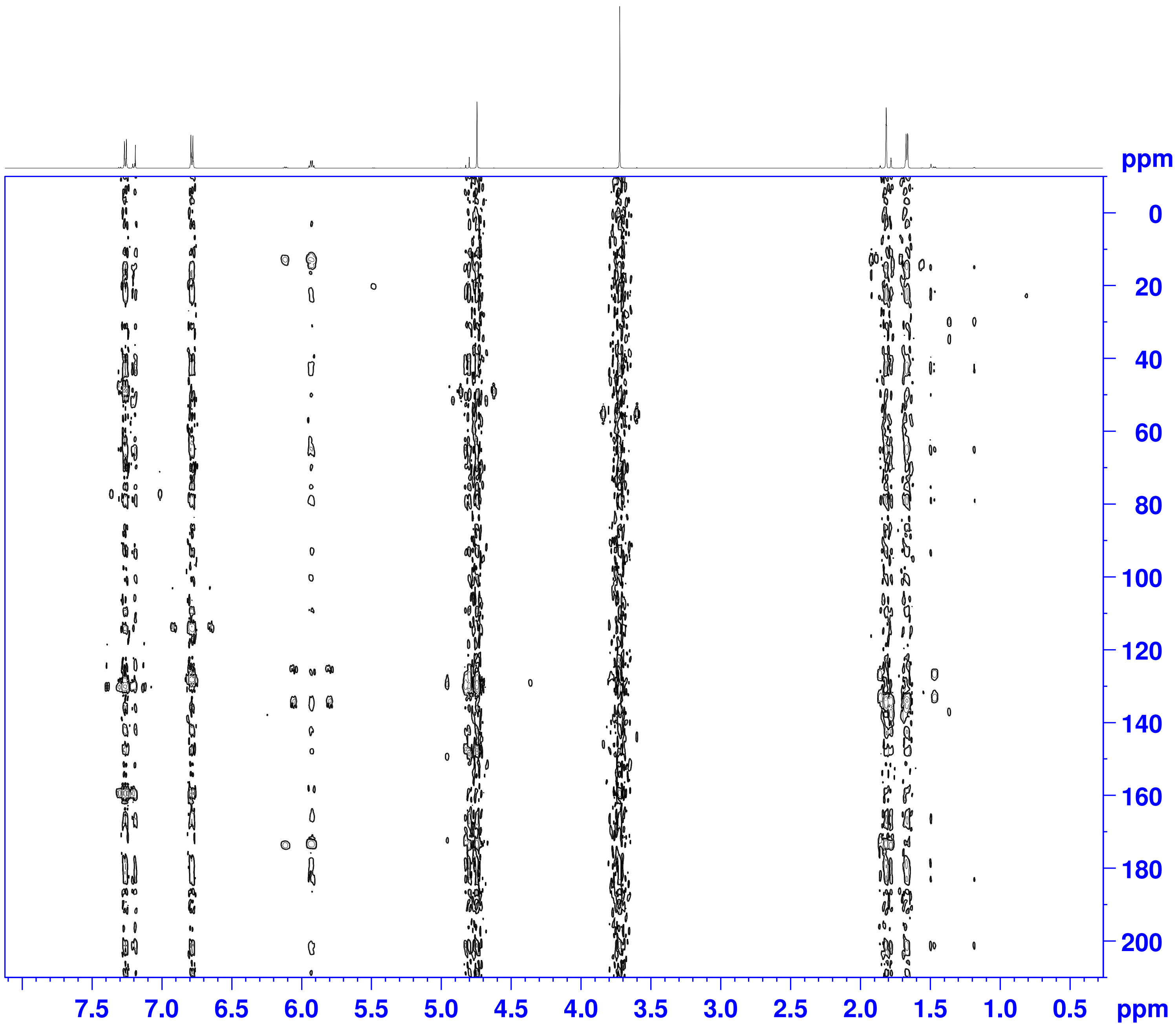


```
Current Data Parameters
NAME      EM06-035-isomer#2-1HMBC
EXPNO                  8
PROCNO                 1

F2 - Acquisition Parameters
Date_           20250212
Time                1.50 h
INSTRUM   Avance Neo 600 at University of Delaware
PROBHD    Z185170_0001 (
PULPROG       hmbclpndqf
TD                  2048
SOLVENT            CDCl3
NS                    32
DS                    16
SWH             4716.981 Hz
FIDRES          4.606427 Hz
AQ             0.2170880 sec
RG                   101
DW               106.000 usec
DE                 10.00 usec
TE                 298.0 K
CNST2        145.0000000
CNST13         8.0000000
D0            0.00000300 sec
D1            1.50000000 sec
D2            0.00344828 sec
D6            0.06250000 sec
IN0           0.00001506 sec
TDav                   1
SFO1         600.1325742 MHz
NUC1                  1H
P1                  8.07 usec
P2                 16.14 usec
PLW1          6.90000010 W
SFO2         150.9178988 MHz
NUC2                 13C
P3                 12.55 usec
PLW2        106.00000000 W

======== F1 INDIRECT DIMENSION ========
td1       128
sw_F1     220.000000

F1 - Acquisition parameters
TD                   128
SFO1            150.9179 MHz
FIDRES        518.780273 Hz
SW               220.000 ppm
FnMODE                QF

F2 - Processing parameters
SI                  2048
SF           600.1300578 MHz
WDW                 SINE
SSB                    0
LB                     0 Hz
GB                     0
PC                  1.40

F1 - Processing parameters
SI                  1024
MC2                   QF
SF           150.9028085 MHz
WDW                 SINE
SSB                    0
LB                     0 Hz
GB                     0
```
